# A TNFR2-Specific TNF Fusion Protein With Improved *In Vivo* Activity

**DOI:** 10.3389/fimmu.2022.888274

**Published:** 2022-06-13

**Authors:** Juan Gamboa Vargas, Jennifer Wagner, Haroon Shaikh, Isabell Lang, Juliane Medler, Mohamed Anany, Tim Steinfatt, Josefina Peña Mosca, Stephanie Haack, Julia Dahlhoff, Maike Büttner-Herold, Carolin Graf, Estibaliz Arellano Viera, Hermann Einsele, Harald Wajant, Andreas Beilhack

**Affiliations:** ^1^Interdisciplinary Center for Clinical Research Laboratory, Department of Internal Medicine II, University Hospital Würzburg, Würzburg, Germany; ^2^Graduate School of Life Sciences, Würzburg University, Würzburg, Germany; ^3^Division of Molecular Internal Medicine, Department of Internal Medicine II, University Hospital Würzburg, Würzburg, Germany; ^4^Department of Microbial Biotechnology, Institute of Biotechnology, National Research Center, Giza, Egypt; ^5^Department of Nephropathology, Friedrich-Alexander-Universität Erlangen-Nürnberg, Erlangen, Germany

**Keywords:** agonist, GvHD, regulatory T cells, serum retention, TNF, TNFR2

## Abstract

Tumor necrosis factor (TNF) receptor-2 (TNFR2) has attracted considerable interest as a target for immunotherapy. Indeed, using oligomeric fusion proteins of single chain-encoded TNFR2-specific TNF mutants (scTNF80), expansion of regulatory T cells and therapeutic activity could be demonstrated in various autoinflammatory diseases, including graft-versus-host disease (GvHD), experimental autoimmune encephalomyelitis (EAE) and collagen-induced arthritis (CIA). With the aim to improve the *in vivo* availability of TNFR2-specific TNF fusion proteins, we used here the neonatal Fc receptor (FcRn)-interacting IgG1 molecule as an oligomerizing building block and generated a new TNFR2 agonist with improved serum retention and superior *in vivo* activity.

**Methods:**

Single-chain encoded murine TNF80 trimers (sc(mu)TNF80) were fused to the C-terminus of an in mice irrelevant IgG1 molecule carrying the N297A mutation which avoids/minimizes interaction with Fcγ-receptors (FcγRs). The fusion protein obtained (irrIgG1(N297A)-sc(mu)TNF80), termed NewSTAR2 (New selective TNF-based agonist of TNF receptor 2), was analyzed with respect to activity, productivity, serum retention and *in vitro* and *in vivo* activity. STAR2 (TNC-sc(mu)TNF80 or selective TNF-based agonist of TNF receptor 2), a well-established highly active nonameric TNFR2-specific variant, served as benchmark. NewSTAR2 was assessed in various *in vitro* and *in vivo* systems.

**Results:**

STAR2 (TNC-sc(mu)TNF80) and NewSTAR2 (irrIgG1(N297A)-sc(mu)TNF80) revealed comparable *in vitro* activity. The novel domain architecture of NewSTAR2 significantly improved serum retention compared to STAR2, which correlated with efficient binding to FcRn. A single injection of NewSTAR2 enhanced regulatory T cell (Treg) suppressive activity and increased Treg numbers by > 300% *in vivo* 5 days after treatment. Treg numbers remained as high as 200% for about 10 days. Furthermore, a single *in vivo* treatment with NewSTAR2 upregulated the adenosine-regulating ectoenzyme CD39 and other activation markers on Tregs. TNFR2-stimulated Tregs proved to be more suppressive than unstimulated Tregs, reducing conventional T cell (Tcon) proliferation and expression of activation markers *in vitro*. Finally, singular preemptive NewSTAR2 administration five days before allogeneic hematopoietic cell transplantation (allo-HCT) protected mice from acute GvHD.

**Conclusions:**

NewSTAR2 represents a next generation ligand-based TNFR2 agonist, which is efficiently produced, exhibits improved pharmacokinetic properties and high serum retention with superior *in vivo* activity exerting powerful protective effects against acute GvHD.

## Introduction

Tumor necrosis factor (TNF) receptor 2 (TNFR2) regulates various facets of the immune system, most notably the activity of pro- and anti-inflammatory cell types including several types of myeloid cells but also cytotoxic T cells (CTLs) and regulatory B and T cells (Bregs, Tregs). TNFR2 acts as a costimulator of CD8^+^ T cells but also promotes Treg expansion and enhances the suppressive effect of Tregs on conventional T cells (1). Therefore, TNFR2 is a promising therapeutic target for the immunotherapy of cancer but also for the treatment of autoimmune syndromes. Accordingly, several groups developed agonists for TNFR2 and tested their activity *in vivo* in models of autoimmunity, neuronal injury and cancer. TNFR2 agonists used are either agonistic antibodies or oligomerized variants of soluble TNF harboring mutations preventing the interaction with TNFR1. Agonistic TNFR2 antibodies typically require binding to Fcγ receptors (Fc*γ*Rs) to display comprehensive robust agonism (2). The FcγR-dependency of the agonism of anti-TNFR2 antibodies, however, not only limits TNFR2 activity by the availability of FcγR-expressing cells *in vivo* but also complicates applicability by unwanted FcγR effector functions (3). Conventional soluble trimeric TNF, in contrast to membrane TNF, does not or only poorly activate TNFR2 (4). Physical connection of two or more soluble TNF trimers by genetic engineering or cross-linking, however, overcomes this limitation and results in TNFR2 agonists with high specific activity *in vitro* (anti-Flag oligomerized Flag-TNF (5); nonameric TNC-scTNF (6); hexameric EDH2-scTNF (7)). Indeed, nonameric and hexameric TNFR2-specific scTNF variants showed therapeutic activity in murine disease models to treat GvHD (8), collagen-induced arthritis (9, 10), experimental autoimmune encephalomyelitis (11, 12) and T cell transfer colitis (13). The currently available formats for oligomeric single-chain (sc) ligand-based TNF receptor agonists show, however, suboptimal serum retention (EDH2 scaffold, 90% loss in < 8 h (14); TNC scaffold, > 99% loss in 12 h, this study).

Here, we describe a novel single-chain ligand based TNFR2-specific agonist, irrIgG1(N297A)-HC:sc(mu)TNF80 termed NewSTAR2, which was obtained by genetic fusion of a trimeric sc(mu)TNF80 domain to the C-terminus of a FcγR-binding defective IgG1 mutant. This novel sc(mu)TNF80 fusion protein was not only efficiently produced but also retained the ability to interact with the neonatal Fc receptor (FcRn) and thus displayed superior serum retention. In fact, a single injection of NewSTAR2 (irrIgG1(N297A)-HC:sc(mu)TNF80) turned out to be sufficient to protect against acute GvHD after allo-HCT.

## Material and Methods

### Cell Lines and Reagents

HEK293T and HT1080-Bcl2-TNFR2 cells were cultivated in RPMI 1640 medium with 10% fetal calf serum (FCS) (both Life technologies, Karlsruhe, Germany). SCF-ER-Hoxb8 immortalized murine myeloid progenitor cells (MPCs) were cultivated and differentiated into macrophages as describe in detail elsewhere (15, 16). HT1080-Bcl2-TNFR2 cells were generated by stable transfection of HT1080-Bcl2(GFP) cells (17) by retroviral transfection with a mixture of a TNFR2 and a puromycin encoding vector. Expression plasmids encoding human FcRn (#RC200364), murine FcRn (MG#205688) and beta-2 microglobulin (#RC207587) were obtained from Origene (Rockville, USA). The expression plasmid encoding murine TNFR2 was purchased from SourceBioscience (Nottingham, United Kingdom). The caspase inhibitor Z-VAD-FMK was obtained from Thermo Fisher Scientific Waltham, MA, USA (Bachem #N-1510.0025). TNC-sc(mu)TNF80 (STAR2) was previously described (8). irrIgG1(N297A)-HC:sc(mu)TNF80 (NewSTAR2) was obtained by transferring the single chain-encoded murine TNF80 trimer (sc(mu)TNF80) domain of TNC-sc(mu)TNF80 to the C-terminus of the human IgG1(N297A) variant of the human CD95-specific antibody E09 (18). Since E09 does not recognize murine CD95 (**Supplementary Figure S1**), it acts as an irrelevant (irr) antibody in mice. The N297A mutation in the IgG1 furthermore prevent/reduce interaction with FcγRs (19). The annotated aa sequences of the heavy and light chain of irrIgG1(N297A)-HC:sc(mu)TNF80 are shown in **Supplementary Figure S2**.

### Production and Purification of STAR2 (TNC-sc(mu)TNF80) and NewSTAR2 (irrIgG1(N297A)-HC:sc(mu)TNF80)

STAR2, NewSTAR2 and the corresponding *Gaussia princeps* luciferase (GpL) fusion proteins all contain a Flag tag (**Supplementary Figure S2**) and were produced by transient transfection of the corresponding expression plasmids in HEK293T cells using polyethylenimine (PEI, Polyscience Inc., Warrington, USA) as described elsewhere in detail (20). In brief, HEK293T cells were incubated with the PEI/DNA-containing transfection medium for one day. The transfection medium was then replaced by RPMI 1640 medium supplemented with 2% FCS. After 5-7 days of cultivation, supernatants with the secreted proteins were harvested, cleared from cellular debris by centrifugation (10 min, 4630 × g) and analyzed by western blotting along with a Flag-tagged protein standard of known concentration to determine productivity. The Flag-tag contained in all proteins was also exploited for purification by affinity chromatography on anti-Flag M2 agarose (Sigma-Aldrich, #A2220) as described by the manufacturer. Purity and concentration of the proteins after purification were evaluated by SDS-PAGE following silver staining of the gel with the Pierce Silver Stain Kit (Thermo Fisher Scientific, MA, USA, 24612) and protein standards of known concentrations and molecular weight (Amersham LMW Calibration Kit for SDS Electrophoresis, GE Healthcare). The LPS content of purified proteins was analyzed with the Pierce LAL Chromogenic Endotoxin Quantitation Kit (Thermo Fisher Scientific) according to the manufacturer’s protocol. If LPS was detected, it was removed using the Pierce High Capacity Endotoxin Removal Resin as described by the manufacturer.

### High-Performance Liquid Chromatography (HPLC)

Purified STAR2 and NewSTAR2 (100 µl) were further analyzed for their native weight and potential protein aggregation by gel filtration on a MabPac SEC-1 column (#088460, Thermo Fisher) using the UltiMate 3000 HPLC system (Thermo Fisher) and the aqueous SEC-1 column performance check standard (#AL0-3042, Phenomenex, Torrance, CA, USA).

### Cellular Binding Studies

HEK293T cells were transiently transfected with expression plasmids encoding murine TNFR2, human FcRn, murine FcRn or empty vector. In case of the two FcRn an expression plasmid encoding beta-2 microglobulin was co-transfected (plasmid ratio 1:1). Next day, TNFR2/FcRn transfected cells and empty vector-transfected cells were harvested, and aliquots were pairwise incubated for 1 h at 37°C in RPMI 1640 medium with 10% FCS (0.5-1 x 10^6^ cells per 1.5 ml tube) with the GpL fusion protein molecules of interest. Unbound GpL fusion protein molecules were removed by three washes with ice-cold PBS (centrifugation 1 min, 14.000 rpm). Cells were then resuspended in 50 µl of RPMI 1640 medium with 0.5% FCS and transferred to a black 96-well plate to quantify the remaining cell-bound GpL fusion protein molecules. After starting the luciferase reaction by adding 25 µl GpL assay solution (1.5 µM coelentrazine (Carl Roth, Germany) in PBS) luciferase activity was immediately measured using a LUmo Luminometer (Anthos Labtec Instruments, Germany). Please note, in the case of the binding studies with the FcRn variants, the pH value of RPMI 1640 medium with 10% FCS and the ice-cold PBS for washing had been adjusted to pH 5.5. Data were analyzed with the “nonlinear regression to a one-site specific binding curve” function of the GraphPad Prism5 software. Specific binding was obtained by subtraction of the non-specific binding values (empty vector) from the cells expressing the receptor of interest (total binding).

### Western Blot

Hoxb8 immortalized murine myeloid progenitor cells (MPCs) were differentiated into macrophages, seeded on 6-well plates (1 × 10^6^ per well) and stimulated as indicated with STAR2 and NewSTAR2. Stimulation was stopped by scratching cells on ice into PBS. After two washes with ice-cold PBS, total cell lysates were mixed with Laemmli buffer and sonificated (UP100H, Helscher, cycle 1, 100% amplitude, 25 s). Total cell lysates were cleared from remaining insoluble material (15 min eppifuge, full-speed) and separated by SDS-PAGE. Proteins were transferred to 0.2 μm nitrocellulose blotting membranes (GE Healthcare Life Sciences) and the latter were blocked with 5% nonfat-dried milk in TBS-Tween (0.05% (v/v)) at room temperature for one hour. Detection of the proteins of interest was accomplished by incubation with appropriate primary antibodies (4°C, overnight, TBS-Tween) and HRP-linked secondary antibodies (room temperature, 1–3 h, 2.5% nonfat-dried milk). Finally, complexes of primary and secondary antibodies were visualized using ECL solution. Antibodies used were purchased from Cell Signaling Technology Beverly, MA, USA: anti-p100/p52 (#4882), TRAF1 (#4715), TRAF2 (#4724), anti-rat IgG HRP-linked antibody (#7077) and anti-rabbit IgG, HRP-linked antibody (#7074). Furthermore, HRP-conjugated polyclonal rabbit anti-mouse immunoglobulin (P0260, Dako), anti-cIAP1 (1E1-1-10, Enzo) anti-β-actin (A1978-200, Sigma Aldrich) were used.

### Viability Assay

Hoxb8 immortalized murine myeloid progenitor cells (MPCs) were differentiated into macrophages (2 × 10^4^ per well), transferred in 96-well plates and stimulated in 100* *μl culture medium with the protein of interest. Cell viability was determined using the MTT assay. For normalization of viability, cells that had been treated with a cytotoxic mixture containing 1* *μg/ml Fc-CD95L 2,5* *μg/ml CHX and sodim azide (0.02 % w/v) defined maximal cell death (0% viability) and cells treated solely with Z-VAD-FMK served to define fully viable control cells (100% viability). Averages of several independent experiments were considered for analysis using GraphPad Prism5 software (GraphPad software, La Jolla, CA, USA).

### ELISA

2 x 10^4^ HT1080-Bcl2-TNFR2 cells were seeded in a 96-well plate. To minimize the background of constitutive IL8 production, medium was changed before stimulation with diluted sera of mice challenged with NewSTAR2, STAR2 or PBS as a control. After overnight incubation, supernatants were analyzed using the IL8 enzyme-linked immunosorbent assay (ELISA) kit from BD Biosciences, Heidelberg, Germany according to the manufacturer’s protocol.

### Mice

C57BL/6 (“B6”) and FVB/N mice were obtained from Janvier Labs (Le Genest Saint Isle, France). Albino C57BL/6J-Tyr^c-2J^/J (“B6a”), albino C57BL/6J-Tyr^c-2J^/Foxp3.Luci.DTR-4 (“FoxP3-Luci-DTR”) (21, 22), albino C57BL/6-Tyr^c-2J^/129S2-Tnfrsf1b^tm1Mwm^/J (“TNFR2-KO”), FVB/N.L2G85 (“FVB-Luci^+^”) and C57BL/6J.L2G85-CD90.1 (“B6-Luci^+^”) (23, 24), were bred at the Center for Experimental Molecular Medicine (ZEMM) in Würzburg, Germany. All mice were housed in the specific pathogen-free facility of the ZEMM, and experiments were carried out according to the German regulations and reviewed and approved by the governmental authorities (Regierung von Unterfranken, 55.2.2-2532-2-537-61 and 55.2.2-2532-2-410-75).

### *In Vitro* Stimulation

Single cell suspensions of enriched CD4^+^ or total T cells were isolated from murine splenocytes through negative isolation magnetic bead enrichment kits (Invitrogen Ref. 11416D or 11413D respectively). 2x10^5^ cells were cultured in 96-well plates for 3 days in complete RPMI (cRPMI) containing 10% FCS, 1% L-Glutamine, 1% Pen/Strep and supplemented with 1,67 nM (450 ng/ml) NewSTAR2 (irrIgG1(N297A)-sc(mu)TNF80), 40 IU recombinant mouse IL-2 (0,882 ng/ml) or PBS.

### *In Vivo* Stimulation, Serum Collection and *In Vivo* Bioluminescence Imaging

For *in vivo* Treg expansion experiments and GvHD studies luciferase-transgenic FoxP3-Luci-DTR mice that express eGFP and luciferase under the FoxP3 promoter (21, 22) or B6 WT mice, respectively, were injected intraperitoneally (i.p.) with 140 µg of NewSTAR2 or the same volume of PBS. For determination of serum retention, mice were injected with STAR2 and NewSTAR2 or PBS i.v. and blood was collected approximately 1-2 min later (defined as t = 0) and after 6, 12 and 24 h from the facial vein. After centrifugation for 10 minutes at 1.500 g at room temperature, sera were collected and stored at -80°C before further use *in vitro*. On the days of *in vivo* imaging, D-Luciferin (300 mg/kg bodyweight) was injected i.p. and mice were anesthetized with 2% isoflurane in oxygen. Images were acquired after 10 min of injection with an IVIS Spectrum CCD-imaging system (Perkin-Elmer) and analyzed with Living image^®^ 4.5.5 software as previously described (25). In brief, exposure time for each picture was 5 minutes with medium binning settings. All images were set at the same scale analyzed together. Bioluminescent signals (p/s/cm^2^/sr) were measured separately from whole mice at defined regions of interest and compared between treatments along the different time points. Relative Treg signals in albino FoxP3-Luci-DTR mice were defined as the percentage increase of bioluminescence signal over the initial signal from day 0 before injection of NewSTAR2 or control antibody subtracting the background signal from that same region of interest. For selected experiments, spleens were dissected 4 days after *in vivo* stimulation and single cell suspensions of splenocytes were analyzed with flow cytometry on an Attune NxT flow cytometer (Invitrogen) and FlowJo software (version 10.6, Tree Star, Ashland, USA).

### Tcon Suppression Assay

Tregs were isolated from spleens of mice, which 4 days before had been i.p. injected with 140 µg of NewSTAR2, through negative isolation magnetic bead enrichment kits (Invitrogen Ref. 11463D, Miltenyi Biotec Ref. 130-091-041). 5x10^4^ Cell-Trace violet (CTV)-labeled total T cells or CD4^+^ T cells were plated together with equal numbers of Tregs (1:1) in V-bottom 96-well plates in cRPMI. For different Tcon : Treg ratios, Tregs were titrated down through serial dilutions to achieve the desired ratios (2:1, 4:1, 8:1) maintaining a maximum of 1x10^5^ cells per well. Activation of Tcons was accomplished by adding a suboptimal concentration (1/4 of the amount specified in the manufacturer’s protocol) of CD3/CD28 activator beads (Invitrogen Ref. 11456D) to each well to avoid overstimulation of cells to facilitate the analysis of Treg suppressive activity. Suppression was assessed based on proliferation of Tcons tracked by dilution of CTV dye after 3 days of coculture at 37°C. As negative control, 5x10^4^ CTV-labeled T cells were plated in cRPMI with 10 ng/ml IL-7. As positive control, 5x10^4^ T cells with activator beads were plated per well.

### Allogeneic HCT

Female B6 mice in the age of 8-12 weeks were myeloablatively irradiated with 9 Gy in a Faxitron CP-160 X-ray system before HCT. 5x10^6^ bone marrow cells and 6x10^5^ T cells (enriched with the aforementioned kit) from female FVB/N or FVB. Luci mice were injected intravenously within 4 hours of total body irradiation to induce acute GvHD.

### Histopathological Evaluation

Organs from euthanized mice were fixed in 4% periodate-lysine-paraformaldehyde, paraffin embedded, sectioned, and stained with hematoxylin and eosin. Slides were examined by an experienced pathologist (M. B.-H.) who was blinded to experimental history. A histological sum score was applied including crypt apoptotic body counts, intensity of inflammation, crypt loss/destruction, giant cells, architectural distortion, apoptotic crypt abscesses and loss of Paneth cells.

### Flow Cytometry

Cell cultures and single cell suspensions were incubated in 5% normal rat serum in PBS for 5 min at 4°C before staining with fluorochrome-conjugated monoclonal antibodies for 30 min at 4°C. Following antibodies were used: anti-CD3 (17A2, Biolegend), anti-CD4 (CRM4-5, Biolegend), anti-CD8 (53-6.7, Biolegend), anti-CD25 (PC61, Biolegend), anti-CD39 (24DMS1, Invitrogen), anti-CD44 (IM7, Biolegend), anti-CD73 (eBioTY/11.8, eBioscience), anti-CD90.1 (Thy1.1; HIS51, eBioscience), anti-CD134 (anti-OX-40; OX-86, eBioscience), anti-CD137 (anti-4-1BB; 17B5, Biolegend), anti-CD223 (anti-LAG-3; eBioC9B7W, eBioscience), anti-CD279 (anti-PD-1; RMP1-30, Biolegend), anti-CD357 (anti-GITR, DTA-1, Biolegend), anti-TIGIT (GIGD7, eBioscience), anti-VISTA (MIH64, Invitrogen). To exclude dead cells from the analysis of live cells by gating, samples were supplemented with the Zombie Aqua Fixable Viability kit. For intracellular anti-Foxp3 (FJK-16s, Invitrogen) and anti-Ki67 (16A8, Biolegend) stainings, cells were fixed with Foxp3/transcription factor Fixation/Permeabilization kit (eBioscience Ref. 00-5521-00) according to the manufacturer’s protocol and then anti-mouse Foxp3-FITC or -APC and/or Ki67-APC conjugated antibodies were added to the permeabilization buffer (1X in deionized water, eBioscience Ref. 00-8333-56) and incubated for 30 min at 4°C before data acquisition. Data were recorded on an Attune NxT cytometer (Thermo Fisher). Compensation of spillover with single staining controls of OneComp eBeads (Thermo Fisher Ref. 01-1111-41) was performed to build compensation matrixes for the different antibody panels. Fluorescence-minus-one method was carried out for the gating of cell subsets. Acquired data were analyzed with FlowJo V10 software.

### Statistical Analyses and Software

Data are shown as mean ± standard deviation (SD). Each data point represents one biological replicate unless stated otherwise, e.g. for experiments were pooled samples are shown. Comparison of means between different groups was based on two-tailed unpaired Student’s t-test. Comparison of mean square residuals between groups was done by one-way ANOVA. Significant differences between groups in survival assays were obtained by Log-Rank test. Level of significance p < 0.05 unless stated otherwise. Statistical tests and graphs were realized with the GraphPad Prism software version 8 or 9. Depicted schemes of experimental set-ups in **Figures 5A, B** were created with BioRender.

## Results

### NewSTAR2 Interacts With the Neonatal Fc Receptor and Has Superior Serum Retention

**Figure 1:**
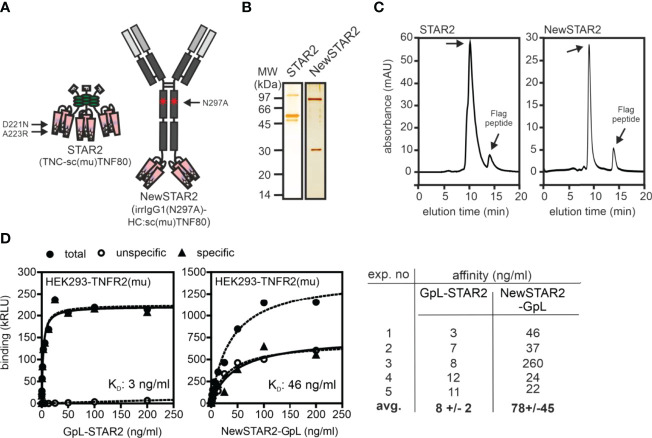
Purification of TNFR2-selective TNF fusion proteins. **(A)** Domain architecture of STAR2 (TNC-sc(mu)TNF80) and NewSTAR2 (irrIgG1(N297A)-HC:sc(mu)TNF80). Please note, aa positions refer to full-length murine TNF (ac. no. AAC82484). **(B, C)** Proteins were purified by anti-Flag affinity chromatography and analyzed by **(B)** SDS-PAGE and **(C)** gel filtration. **(D)** Binding of GpL fusion proteins of STAR2 and NewSTAR2 to HEK293 cells transfected with empty vector or a murine TNFR2 expression plasmid. Specific binding was calculated as the difference between total binding (TNFR2 transfectants) and unspecific binding (empty vector transfectants). Left and middle panel: representative experiments; right panel summarizing table of 5 independent experiments.

**Figure 2:**
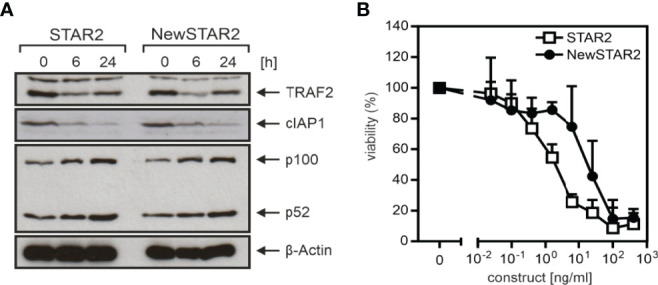
*In vitro* activity of TNFR2-selective muTNF fusion proteins. **(A)** Macrophages differentiated from Hoxb8 immortalized murine MPCs were stimulated overnight with 200 ng/ml of STAR2 or NewSTAR2 and total cell lysates were analyzed by western blotting for the presence of the indicated proteins. One representative experiment of 3 is shown. **(B)** Macrophages were stimulated with the indicated concentrations of STAR2 or NewSTAR2 in the presence of 20 μM Z-VAD-FMK. After 36 h cell viability was evaluated. Data shown are averaged from 3 independent experiments.

**Figure 3:**
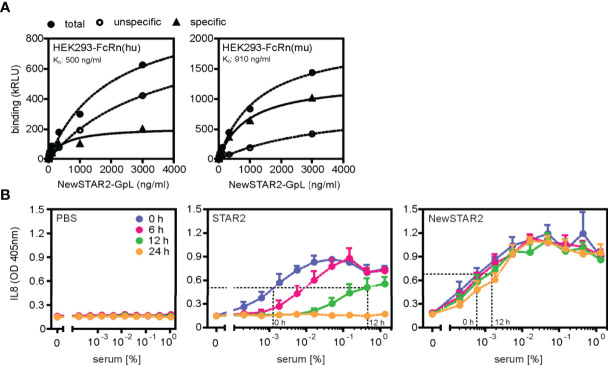
NewSTAR2 has a superior bioavailability. **(A)** Specific binding of a GpL fusion protein of NewSTAR2 to cells expressing the human or murine neonatal Fc receptor (FcRn). **(B)** Mice (3 per group) were injected i.v. with 50 μg STAR2 or 75 μg NewSTAR2 or PBS. Blood was taken at indicated time points after treatment, diluted as indicated and used to stimulate HT1080-Bcl2-TNFR2 cells. IL-8 in the supernatant was determined the next day to quantify TNFR2 engagement. The dashed y-lines show the level of half-maximal IL8 induction to facilitate identification of EC_50_-values.

To improve the serum retention of TNC-sc(mu)TNF80 (STAR2; selective TNF-based agonist of TNF receptor 2 (8)), we transferred its single chain-encoded TNF80 trimer domain to the C-terminus of the heavy chain of an IgG molecule (**Figure 1A**). For this purpose, we used the human IgG1 antibody E09, which recognizes human CD95 (18) but not its murine counterpart (**Supplementary Figure S1**) and which in mice can therefore be considered as an antibody of irrelevant specificity. To prevent/reduce interaction with FcγRs, we furthermore introduced the N297A mutation into the IgG1 scaffold (19). The resulting fusion protein irrIgG1(N297A)-HC:sc(mu)TNF80, in the following also designated as NewSTAR2 (New selective TNF-based agonist of TNF receptor 2), was better produced than STAR2 (with 10 µg/ml versus 2 µg/ml and gel filtration of the purified protein gave no evidence for unwanted aggregation (**Figures 1B, C**). Using variants of STAR2 and NewSTAR2, in which the *Gaussia princeps* luciferase (GpL) had been genetically linked as an easily quantifiable reporter domain to the N-terminus of STAR2 and the C-terminus of the NewSTAR2 light chain, respectively, cellular binding studies were performed to determine affinity for murine TNFR2. Affinities obtained were in average 8 ng/ml (44 pM) for GpL-STAR2, which is close to previous results (8), and in average 78 ng/ml (390 pM) for NewSTAR2-GpL (**Figure 1D****)**. The lower apparent affinity of NewSTAR2 compared to STAR2 may reflect the higher avidity of STAR2 which contains three sc(mu)TNF80 modules while NewSTAR2 has only two (**Figure 1A**). Next, we evaluated in macrophages differentiated from HoxB8 immortalized myeloid progenitor cells the ability of NewSTAR2 versus STAR2 to trigger the alternative NF-κB pathway and to induce cell death in the presence of compromised caspase-8 activity. Both, STAR2 and NewSTAR2 efficiently triggered the alternative NF-κB pathway evident from degradation of TRAF2 and cIAP1 and enhanced p100 processing (**Figure 2A**). Moreover, NewSTAR2 was almost as efficient as STAR2 in inducing cell death in macrophages treated with the caspase inhibitor Z-VAD-FMK (**Figure 2B**). The expectation that NewSTAR2 might display superior serum retention is based on the ability of IgG1 molecules to interact with the FcRn. Indeed, using the GpL fusion protein variant of NewSTAR2, we found that the genetic fusion of the sc(mu)TNF80 domain to the C-terminus of the IgG1(N297) heavy chain left intact the ability of the IgG1 scaffold to interact with the human and murine FcRn (**Figure 3A**). Accordingly, we observed strongly enhanced serum retention of NewSTAR2 compared to STAR2 when we analyzed blood taken from mice different times after i.v. injection of the two TNFR2 agonists (**Figure 3B**). For example, immediately after injection (defined as t = 0 h) the sera of mice injected with STAR2 and NewSTAR2 enabled half-maximal TNFR2-mediated IL8 induction *in vitro* at comparable concentrations of approximately 0.001% (**Figure 3B**). Corresponding sera of blood samples taken 12 hours post injection, however, revealed strikingly different activities. The NewSTAR2-containing 12 h sera showed only a minor approximately 2-fold reduction in their IL8 inducing activity (EC_50_ value 0.002%). In contrast, the activity of the STAR2-containing 12 h sera dropped about 400-fold to approx. 0.4% (**Figure 3B**), thus, under the assumption of a constant exponential decay, this argues for a half-life of < 1.5 h.

In sum, our data indicate that NewSTAR2 is an efficient TNFR2 agonist comparable to STAR2 *in vitro*. Yet, NewSTAR2 features superior productivity and strongly improved serum retention. Furthermore, we obtained similar results with a NewSTAR2-related molecule that is based on murine IgG1(D265A) instead of human IgG1(N297A) (**Supplementary Figure S3**).

### NewSTAR2 Efficiently Expands Regulatory T Cells *In Vivo*

**Figure 4:**
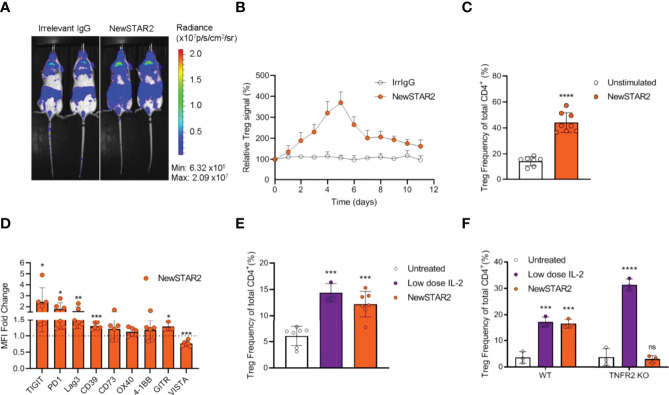
NewSTAR2 expands Tregs *in vivo*. **(A)** B6a.FoxP3.LuciDTR female mice were i.p. injected with 140 µg of NewSTAR2 or control IgG1 in PBS on day 0. Pictures from 2 representative mice of each group imaged on day 4 after NewSTAR2 injection. **(B)** Averaged relative increase in bioluminescence signal derived from luciferase expressing FoxP3^+^ Tregs compared to the ventral signal of each mouse before treatment (d0). NewSTAR2 n=6; Irrelevant IgG1 n=8. **(C)** Relative CD4^+^FoxP3^+^ Treg frequencies in spleens isolated from mice on day 4 after stimulation measured with flow cytometry; n=5/group. **(D)** MFI fold change of distinct Treg markers normalized to the MFI of untreated Tregs. n=6. **(E)** Relative Treg frequencies within splenic CD4^+^ T cells 4 days after *in vitro* treatment; untreated n=7; low dose IL-2 n=3; NewSTAR2 n=7. **(F)** Relative Treg frequencies in CD4^+^ T cells from B6 or B6.TNFR2-KO splenic T cell cultures 4 days after *in vitro* stimulation; n=3. Data are shown as mean +/- SD, *p ≤ 0.05, **p ≤ 0.01, ***p ≤ 0.001, ****p ≤ 0.0001; ns, not significant; two-tailed unpaired Student’s t-test was applied for **(A–D)**. ANOVA was applied to compare untreated cells against NewSTAR2 and low dose IL-2 shown in **(E, F)**.

Previously, we had demonstrated that repeated injections of STAR2 over a period of two weeks expanded Tregs approx.1.5 fold *in vivo* in B6.FoxP3-LuciDTR mice (8). In contrast, already a single NewSTAR2 injection in B6.FoxP3-LuciDTR reporter mice that express luciferase under the FoxP3 promoter induced a strong increase in the bioluminescence signal reaching a >3-fold stronger signal peak over steady-state levels after 4-5 days and a >2-fold increase lasting for over 10 days (**Figures 4A, B**). Accordingly, at the peak time point (4 days) the relative CD4^+^FoxP3^+^ Treg frequency increased roughly 2.5-fold within the splenic CD4^+^ T cell population (**Figure 4C** and **Supplementary Figure 4A, B**). Furthermore, a single injection of NewSTAR2 upregulated various suppression-related markers on splenic Tregs *in vivo* 4 days after treatment. Upregulated surface receptors included the adenosine-regulating ectoenzyme CD39, immune checkpoint molecules such as Programmed cell death protein-1 (PD-1, CD279), T cell immunoreceptor with Ig and ITIM domains (TIGIT) and Lymphocyte-activation gene 3 (Lag3, CD223) as well as glucocorticoid-induced TNFR-related protein (GITR, TNFRSF18, CD357) (**Figure 4D** and **Supplementary Figures S4C,D**). Notably, there were no changes in the frequencies of FoxP3^-^ conventional CD4^+^ T cells (CD4^+^ T cons) and conventional CD8^+^ T cells (CD8^+^ Tcons) (**Supplementary Figure S4A**). Together, these results suggest that the strongly improved serum retention of NewSTAR2 indeed translates into improved *in vivo* TNFR2 agonism.

### Treg Expansion *via* NewSTAR2 Depends on TNFR2

*In vitro* stimulation of negatively enriched splenic CD4^+^ T cells with NewSTAR2 increased Treg frequencies by 2-fold (**Figure 4E**) similar to treatment with low dose IL-2. Increasing numbers of Ki67^+^ Tregs but not of Ki67^+^ Tcons corroborated the specific Treg expansion (**Supplementary Figure S4**). To confirm that the observed effects were mediated *via* TNFR2, we stimulated splenic CD4^+^ T cells *in vitro* from either wild type or TNFR2-KO donors with NewSTAR2 and compared this to low dose IL-2 treatment. Indeed, NewSTAR2 expanded only Tregs from wild type donors similar to low dose IL-2. In contrast, NewSTAR2 could not expand Tregs from splenic TNFR2-KO CD4^+^ T cells whereas low dose IL-2 could (**Figure 4F**).

### Prophylactic Treg Expansion Protects Against Acute GvHD

**Figure 5:**
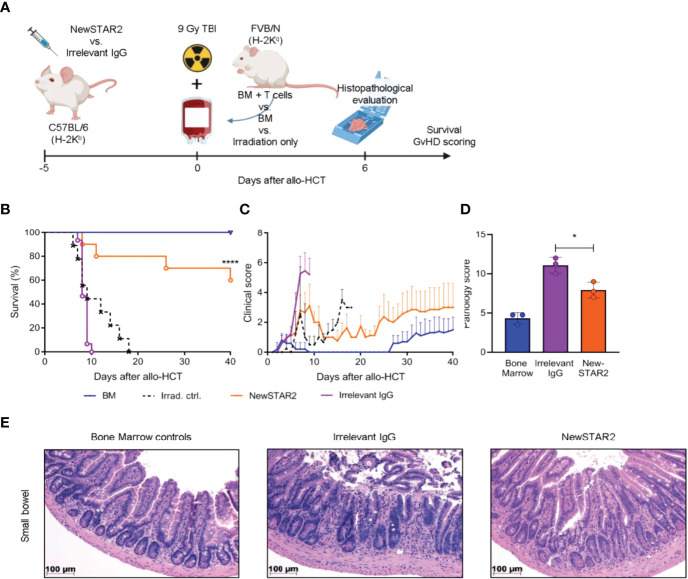
Preemptive Treg expansion with NewSTAR2 protects mice from acute GvHD. **(A)** Experimental procedure of allo-HCT. C57BL/6 or albino C57BL/6J-Tyr^c-2J^/J wild type female mice were injected i.p. with 140 µg of NewSTAR2 or control Irrelevant IgG1 in PBS 5 days before transplantation. 5x10^6^ bone marrow (BM) cells from female FVB/N mice plus 6x10^5^ splenic T cells from either FVB/N or FVB. Luci^+^ female mice were i.v. injected in each myeloablatively irradiated (9 Gy) B6 recipient. Bone marrow control mice were injected only with 5x10^6^ BM cells. Mice for survival experiments were weighed and scored daily for 40 days after allo-HCT. Irradiation control n=9; Bone marrow control n=10; Irrelevant IgG1 control n=15; NewSTAR2 n=10. **(B)** Kaplan-Meier survival graphs. ****p ≤ 0.0001. **(C)** Clinical score of mice. **(D)** Histopathological score at day +6 after allo-HCT. *p ≤ 0.05. **(E)** Representative hematoxylin and eosin stainings of the small bowel at day +6 after allo-HCT.

Previously, we had demonstrated that repeated injections of STAR2 can protect from subsequent allo-HCT-induced acute GvHD in a Treg-dependent manner (8). To test whether NewSTAR2-induced Treg expansion would also translate into a clinical benefit, we employed a model of MHC-major mismatch FVB/N (H-2^q^)→C57Bl/6 (H-2^b^) allo-HCT. On day -4 before allo-HCT, we preemptively treated C57Bl/6 recipients with one i.p. injection of either NewSTAR2 or irrelevant IgG1 as an isotype control (**Figure 5A**). On day 0 these recipient mice were transplanted with allogeneic bone marrow (BM) and T cells to induce acute GvHD. NewSTAR2 treatment improved survival and reduced disease development (**Figure 5B, C**). The single NewSTAR2 treatment extended the median survival time from 9 to 40 days (**Figure 5B**) compared to isotype controls. NewSTAR2 treatment markedly improved clinical scores and allo-HCT recipients developed only mild symptoms during the first 20 days post allo-HCT, which increased until the end of the experiment in some mice but for the majority never reached the humane endpoint that would require to precautionary euthanize animals (**Figure 5C**). Recipients transplanted with splenic T cells and BM that had been treated with irrelevant IgG1 displayed an increased histopathologic GvHD sum score on day 6 after allo-HCT. In contrast, allo-HCT recipients of NewSTAR2-treated T cells and BM showed a significantly lower histopathological sum score (**Figure 5D**). In the lamina propria of controls sparse round cell infiltrates are observed between the crypts without significant expansion of the space between neighboring crypts, whereas in the example of a mouse treated with irrelevant IgG the intercryptic space is expanded by inflammatory cells. This effect is reduced in the NewSTAR2 treated animal depicted in **Figure 5E** without displacement of crypts by inflammation. Thus, a single injection of NewSTAR2 five days prior allo-HCT sufficed to preemptively expand Tregs *in vivo* to reduce experimentally induced acute GvHD in recipient mice.

### NewSTAR2 Enhances the Capacity of Tregs to Suppress CD4^+^ and CD8^+^ Tcons

**Figure 6:**
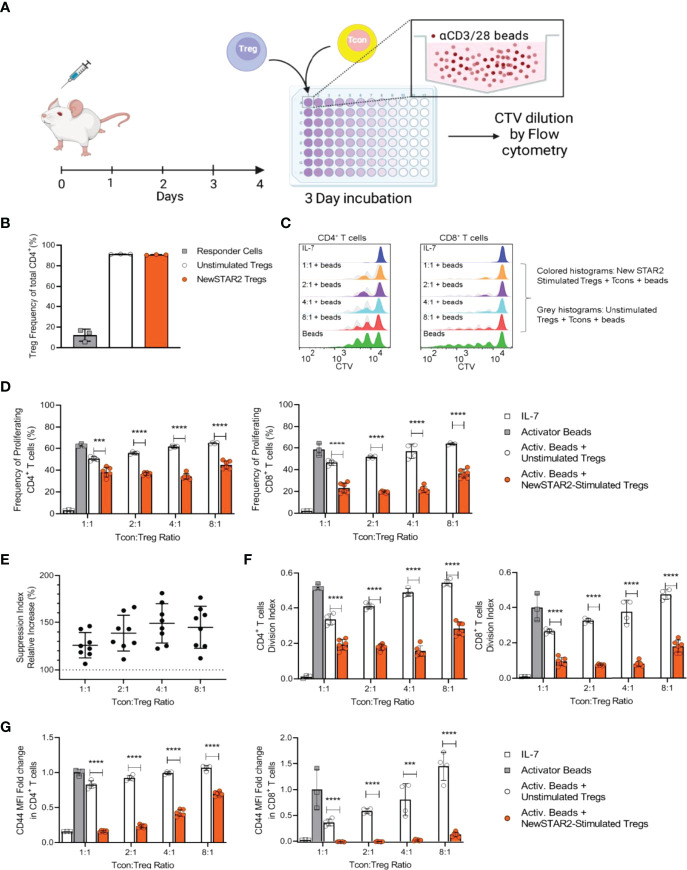
NewSTAR2 increases the suppressive capacity of Tregs. **(A)** Experimental set-up of the *in vitro* Treg suppression assay. B6 wild type mice (CD90.2) were i.p. injected with 140 µg of NewSTAR2 or control Irrelevant IgG1 in PBS and spleens were dissected on day 4 after stimulation. Tregs were enriched through magnetic separation. In parallel, total T cells were isolated from spleens from untreated congenic B6.CD90.1 mice and labeled with CTV dye to measure Tcon proliferation (In repeated experiments CD90.1 Treg donor mice and CD90.2 total T cell donor mice were used). 5x10^4^ Tcons were plated in each well with equal numbers of Tregs or fewer in order to achieve Tcon : Treg ratios of 1:1, 2:1, 4:1 and 8:1. Cells were cultured in cRPMI with suboptimal concentrations of CD3/28 activator beads for 4 days until flow cytometric analysis. **(B)** Representative data of relative Treg purities after cell enrichment. For each set of assays, samples were pooled from 3 mice within each group, and each data point represents one replicate. **(C)** Representative histograms of the responder CD4^+^ (left) and CD8^+^ T cells (right) 4 days after culture. Dark blue histograms: undivided cells cultured with IL-7; green: responder cells cultured with CD3/28 activator beads only. Orange, magenta, light blue and pink histograms show the generations of divided responder T cells by dilution of the CTV dye when cultured with Tregs from NewSTAR2-stimulated mice at the indicated Tcon : Treg ratios. Overlaid gray histograms represent the generations of divided responder T cells by dilution of the CTV dye when cultured with unstimulated Tregs at the indicated Tcon : Treg ratios. **(D)** Frequencies of proliferating CD4^+^ (left) and CD8^+^ responder T cells (right). Each data point represents the averaged values for one set of experiments in which cells of 3 mice were pooled for NewSTAR2-stimulated or unstimulated groups. C and D show representative data from 4 different experiments. **(E)** Increase in responder T cell suppression as the quotient of the frequency of proliferating cells in presence of unstimulated Tregs over the frequency of proliferating cells in presence of NewSTAR2-stimulated Tregs. Data from CD4^+^ and CD8^+^ T cells are shown together for each given ratio. **(F)** Average number of cell divisions that responder T cells of the original population have undergone (= division index) for CD4^+^ (left) and CD8^+^ (right) responder T cells. Representative data from 4 different experiments are shown. **(G)** Fold change of CD44 mean fluorescence expression levels of CD4^+^ T cells normalized to the MFI values of responder cells cultured only with CD23/28 activator beads. **(D–G)** One data point corresponds to the averaged values from each treatment of one suppression assay. Data are presented as means +/- SD, ***p ≤ 0.001, ****p ≤ 0.0001, two-tailed unpaired Student’s *t*-test.

As TNFR2 activation cannot only expand the Treg pool but also increases suppressive Treg activity (1, 26), we asked whether *in vivo* treatment with NewSTAR2 would enhance the suppressive capacity of Tregs on a single cell basis. To address this, we treated B6 mice with a single dose of NewSTAR2 and isolated splenic FoxP3^+^ Tregs four days later (**Figure 6A**). Tregs derived of the NewSTAR2-treated mice and Tregs from untreated donors were then added to an *in vitro* suppression assay with Tcons (FoxP3^-^ CD4^+^ and CD8^+^ T cells) that we activated with αCD3/αCD28 beads (**Figure 6A**). CD90.1 as congenic marker allowed us to distinguish the added Tregs from the αCD3/αCD28 bead-stimulated CD4^+^ and CD8^+^ Tcons. Treg enrichment resulted in a > 90% FoxP3^+^CD4^+^ Treg purity, whereas enriched Tcons contained up to 15% Tregs in the CD4^+^ responder cell fraction, indicating normal Treg relative numbers in responder cells and thus negligible effect from Tregs within the responder population (**Figure 6B**). We chose experimental settings that would not fully inhibit the initial Tcon proliferation upon αCD3/αCD28 bead-stimulation. Conversely, αCD3/αCD28 beads alone did not increase Treg numbers upon *in vitro* cell culture. Accordingly, dilution of the Cell-Trace violet signal by half upon each cell division revealed distinct generations of dividing Tcons that appear as discrete peaks in the flow cytometry histograms (**Figure 6C**). Tregs from NewSTAR2-treated mice suppressed CD4^+^ Tcon proliferation more efficiently than equal numbers of Tregs from untreated donors (**Figure 6D**). The suppressive capacity of Tregs from NewSTAR2-treated donors exceeded Tregs from untreated donors by about 25% and even up to 50% at lower Treg numbers (**Figure 6E**). Proliferation analysis also revealed that NewSTAR2 stimulated Tregs reduced the number of dividing cells rather than the number of divisions (**Figure 6F**). Analogous to the frequency of proliferating cells, NewSTAR2-stimulated Tregs reduced the expression of the activation marker CD44 on Tcons stronger than Tregs from non-treated donor mice (**Figure 6G**). Thus, these data demonstrate that *in vivo* NewSTAR2 treatment also enhances the suppressive capacity of Tregs on a cellular level.

## Discussion

TNFR2 plays a crucial role for human and mouse CD4^+^FoxP3^+^ Treg biology. Stimulation of TNFR2 maintains, activates, expands and stabilizes Tregs in inflammatory conditions and defines highly suppressive Tregs (8, 27–35). In previous proof-of-concept work, we demonstrated that STAR2, a nonameric TNFR2-specific variant of mouse TNF, could not only safely expand Tregs *in vivo* but also protect allo-HCT recipients from acute GvHD while sparing anti-lymphoma and anti-infectious properties of transplanted allogeneic Tcons (8). However, for clinical translation, this original approach had a few technical limitations. Firstly, although STAR2 proved as a potent agonist for TNFR2 *in vitro*, its molecular architecture results in a short *in vivo* half-life of < 1.5 hours (**Figure 3B**) and thus requires repetitive dosing (8). Secondly, the nonameric design of STAR2 suffered from low-yield production requiring considerable efforts to generate sufficient amounts of this agonist for *in vivo* applications. Here we describe a rational approach to overcome these challenges by designing, developing and characterizing NewSTAR2 as a selective and potent TNFR2 agonist *in vitro* and *in vivo*.

To ensure specific binding of TNFR2, reliable receptor clustering of TNFR2 on target cells and improved pharmacokinetic properties to increase serum retention, we combined four protein engineering strategies to construct NewSTAR2. First, in analogy to STAR2, we used a murine TNF protomer preventing TNFR1 binding, thus a TNF mutant with selectivity for TNFR2. Second, we exploited an IgG1 backbone to improve production efficiency and to enable interaction with the FcRn to improve serum retention. Third, we introduced the N297A mutation into the IgG1 backbone to prevent/minimize unwanted FcγR interactions of NewSTAR2. Therefore, NewSTAR2 neither requires Fc receptor binding for activity nor interacts with FcγRs, what could even render TNFR2 activation more complicated *in vivo*, e.g. by triggering FcγR-mediated effector functions. In the worst-case scenario, this could even lead to FcγR-mediated depletion of TNFR2-expressing Tregs instead of selectively stimulating them. Fourth, and last but not least, linking the three protomers containing sc(mu)TNF80 domain to the C-terminus of the IgG1 backbone resulted in a six protomer containing molecule to mimic the known “activating” effect of cross-linking on poorly active TNF trimers. Therefore, NewSTAR2 does not only mimic the strong TNFR2 stimulatory activity of membrane TNF and oligomerized soluble TNF, but also overcomes the moderate productivity and the limited serum retention of STAR2 (**Figures 2**, **3**). It is worth mentioning that the positioning of the sc(mu)TNF80 domain on the IgG1 backbone is largely irrelevant for activity. Initially, we also investigated variants in which the variable domains of the heavy and light chain of an IgG1 had been replaced by the sc(mu)TNF80 domain (sc(mu)TNF80-IgG1(N297A)) or where the latter had been linked to the C-terminus of the light chain of the IgG1 scaffold (irrIgG1(N297A)-LC:sc(mu)TNF80) (**Supplementary Figure S5A**). These constructs are *in vitro* comparably active as NewSTAR2 and STAR2 (**Supplemental Figures S5B,C**) but were not investigated in the follow up studies *in vivo*. Intriguingly, despite having four instead of only two sc(mu)TNF80 domains, sc(mu)TNF80-IgG1 showed no higher activity than NewSTAR2 resembling the fact that TNC-sc(mu)TNF80 (=STAR2) with three such domains are also not much more active than NewSTAR2. Thus, two trimeric scTNF80 domains seem to be necessary but also largely sufficient to ensure high/maximal agonism of oligovalent sc(mu)TNF80 fusion proteins. For the preclinical studies presented here, we used a human CD95-recognizing IgG1(N297) antibody, which was effectively produced in previous work (2), as basis. For the potential translational development of NewSTAR2, however, an antibody with an in men irrelevant specificity has to be used or the sole Fc domain of a FcRn-interacting IgG molecule. In sum, the use of an IgG scaffold in combination with a single-chain encoded TNFR2-specific ligand trimer brings together the pharmacological advantages of an antibody with the high intrinsic activity of oligomerized TNF ligand trimers.

TNFR2 has been recognized as a promising therapeutic target for a broad range of disease conditions, from autoimmune diseases over neurodegenerative diseases, tissue injury and alloimmune responses, such as GvHD, to cancer (7–12, 36–44). So far, several TNFR2-specific agonists have been reported, although most of them have been only studied *in vitro* (34, 45–48). Robust agonistic TNFR2 activation *in vivo* not only requires the selective TNFR2 clustering on target cells, which can be achieved by oligomerized TNFR2-specific TNF trimers but also appropriate pharmacological properties. Indeed, multimeric ligand-based agonists proved as efficient agonists *in vitro* and *in vivo* (8–13, 49). Yet, the major limitation of these agonists is the rather short serum half-lives of a very few hours. In contrast, after 12 hours NewSTAR2 activity decreased by only approx. 2-fold in the serum of mice. NewSTAR2 proved active for days as single injection sufficed to increase Treg numbers for more than 10 days and, importantly, protected from lethal acute GvHD. It is important to note that TNFR2 (and also TNFR1) can induce TNF especially in T-cells and myeloid cells (1). TNFR2 agonists as NewSTAR2 are therefore quite capable to indirectly stimulate TNFR1. However, the fact that NewSTAR2 and other TNFR2 agonist used in the literature do not trigger sepsis like symptoms, the major effect of TNF administration, this possibility is systemically obviously irrelevant. With STAR2 we showed furthermore previously that TNFR2 expands Tregs independently from endogenous TNF and TNFR1 (8). Thus, NewSTAR2 appears as an attractive TNFR2 agonist for clinical applications and awaits to be tested in a broad variety of inflammatory and degenerative disease conditions as well as cancer. Some of the most interesting potential applications for TNFR2 agonists in general and NewSTAR2 in particular are neuronal disorders. Indeed, TNFR2 agonists showed beneficial activity in EAE and preclinical models of spinal cord injury, chronic neuropathic pain and acute neurodegeneration (7, 11, 12, 50, 51). An issue of special relevance is here the potential blood brain barrier (BBB) penetrance of a TNFR2 agonist. In general, the BBB penetrance of antibodies and antibody variants is poor (52, 53). However, this limitation can be overcome, or at least be reduced, by receptor-mediated transcytosis, thus by targeting BBB transporters such as the insulin receptor (IR) the transferrin receptor (TfR) with antibody fusion proteins (52, 53). Therefore, since the specificity of the IgG1(N297A) part, or more general the IgG domain, of NewSTAR2 is irrelevant for its TNFR2-stimulating activity, it is tempting to speculate that BBB penetrance of NewSTAR2 could be enhanced by exploiting IR- or TfR-specific antibodies as IgG scaffold for the NewSTAR2 molecule.

## Data Availability Statement

The raw data supporting the conclusions of this article will be made available by the authors, without undue reservation.

## Ethics Statement

The animal study was reviewed and approved by Regierung von Unterfranken, 55.2.2-2532-2-537-61 and 55.2.2-2532-2-410-75.

## Author Contributions

JW, IL, JM, and MA performed, analyzed and interpreted binding studies and all *in vitro* experiments with cell lines. JG, HS, TS, MP, SH, JD, EV, and CG performed, analyzed and interpreted experiments with primary cells and animals. MB-H performed histopathologic tissue analyses. AB, HE, and HW designed experiments. JG, JW, HS, IL, HW, and AB wrote the manuscript. All authors contributed to the article and approved the submitted version.

## Funding

This work was supported by the Deutsche Forschungsgemeinschaft (DFG, German Research Foundation) – project number 324392634 – TRR 221 (grants to HW (project B02), AB (project B09) and MB-H (project Z01)).

## Conflict of Interest

The University of Würzburg has filed a patent concerning the construction of TNFR2 agonists.

The authors declare that the research was conducted in the absence of any commercial or financial relationships that could be construed as a potential conflict of interest.

## Publisher’s Note

All claims expressed in this article are solely those of the authors and do not necessarily represent those of their affiliated organizations, or those of the publisher, the editors and the reviewers. Any product that may be evaluated in this article, or claim that may be made by its manufacturer, is not guaranteed or endorsed by the publisher.

## References

[1] Wajant H Beilhack A. Targeting Regulatory T Cells by Addressing Tumor Necrosis Factor and Its Receptors in Allogeneic Hematopoietic Cell Transplantation and Cancer. Front Immunol (2019) 10:2040. doi: 10.3389/fimmu.2019.02040 31555271 PMC6724557

[2] Medler J Nelke J Weisenberger D Steinfatt T Rothaug M Berr S. TNFRSF Receptor-Specific Antibody Fusion Proteins With Targeting Controlled Fcγr-Independent Agonistic Activity. Cell Death Dis (2019) 10(3):224. doi: 10.1038/s41419-019-1456-x 30833543 PMC6399339

[3] Medler J Wajant H. Tumor Necrosis Factor Receptor-2 (TNFR2): An Overview of an Emerging Drug Target. Expert Opin Ther Targets (2019) 23(4):295–307. doi: 10.1080/14728222.2019.1586886 30856027

[4] Grell M Douni E Wajant H Löhden M Clauss M Maxeiner B. The Transmembrane Form of Tumor Necrosis Factor is the Prime Activating Ligand of the 80 kDa Tumor Necrosis Factor Receptor. Cell (1995) 83(5):793–802. doi: 10.1016/0092-8674(95)90192-2 8521496

[5] Schneider P Holler N Bodmer JL Hahne M Frei K Fontana A. Conversion of Membrane-Bound Fas(CD95) Ligand to Its Soluble Form Is Associated With Downregulation of Its Proapoptotic Activity and Loss of Liver Toxicity. J Exp Med (1998) 187(8):1205–13. doi: 10.1084/jem.187.8.1205 PMC2212219 9547332

[6] Rauert H Wicovsky A Müller N Siegmund D Spindler V Waschke J. Membrane Tumor Necrosis Factor (TNF) Induces P100 Processing *via* TNF Receptor-2 (TNFR2). J Biol Chem (2010) 285(10):7394–404. doi: 10.1074/jbc.M109.037341 PMC2844188 20038584

[7] Dong Y Fischer R Naudé PJW Maier O Nyakas C Duffey M. Essential Protective Role of Tumor Necrosis Factor Receptor 2 in Neurodegeneration. Proc Natl Acad Sci USA (2016) 113(43):12304–9. doi: 10.1073/pnas.1605195113 PMC5087045 27791020

[8] Chopra M Biehl M Steinfatt T Brandl A Kums J Amich J. Exogenous TNFR2 Activation Protects From Acute GvHD *via* Host T Reg Cell Expansion. J Exp Med (2016) 213(9):1881–900. doi: 10.1084/jem.20151563 PMC4995078 27526711

[9] Fischer R Proske M Duffey M Stangl H Martinez GF Peters N. Selective Activation of Tumor Necrosis Factor Receptor II Induces Antiinflammatory Responses and Alleviates Experimental Arthritis. Arthritis Rheumatol (2018) 70(5):722–35. doi: 10.1002/art.40413 29342501

[10] Lamontain V Schmid T Weber-Steffens D Zeller D Jenei-Lanzl Z Wajant H. Stimulation of TNF Receptor Type 2 Expands Regulatory T Cells and Ameliorates Established Collagen-Induced Arthritis in Mice. Cell Mol Immunol (2019) 16(1):65–74. doi: 10.1038/cmi.2017.138 29375132 PMC6318277

[11] Fischer R Padutsch T Bracchi-Ricard V Murphy KL Martinez GF Delguercio N. Exogenous Activation of Tumor Necrosis Factor Receptor 2 Promotes Recovery From Sensory and Motor Disease in a Model of Multiple Sclerosis. Brain Behav Immun (2019) 81:247–59. doi: 10.1016/j.bbi.2019.06.021 PMC6754799 31220564

[12] Ronin E Pouchy C Khosravi M Hilaire M Grégoire S Casrouge A. Tissue-Restricted Control of Established Central Nervous System Autoimmunity by TNF Receptor 2–Expressing Treg Cells. PNAS (2021) 118(13):e2014043118. doi: 10.1073/pnas.2014043118 33766913 PMC8020675

[13] Lubrano di Ricco M Ronin E Collares D Divoux J Grégoire S Wajant H. Tumor Necrosis Factor Receptor Family Costimulation Increases Regulatory T-Cell Activation and Function *via* NF-κb. Eur J Immunol (2020) 50(7):972–85. doi: 10.1002/eji.201948393 PMC7383872 32012260

[14] Seifert O Plappert A Fellermeier S Siegemund M Pfizenmaier K Kontermann RE. Tetravalent Antibody-scTRAIL Fusion Proteins With Improved Properties. Mol Cancer Ther (2014) 13(1):101–11. doi: 10.1158/1535-7163.MCT-13-0396 24092811

[15] Wang GG Calvo KR Pasillas MP Sykes DB Häcker H Kamps MP. Quantitative Production of Macrophages or Neutrophils *Ex Vivo* Using Conditional Hoxb8. Nat Methods (2006) 3(4):287–93. doi: 10.1038/nmeth865 16554834

[16] Siegmund D Kums J Ehrenschwender M Wajant H. Activation of TNFR2 Sensitizes Macrophages for TNFR1-Mediated Necroptosis. Cell Death Dis (2016) 7(9):e2375. doi: 10.1038/cddis.2016.285 27899821 PMC5059883

[17] Kreuz S Siegmund D Rumpf J-J Samel D Leverkus M Janssen O. NFkappaB Activation by Fas Is Mediated Through FADD, Caspase-8, and RIP and is Inhibited by FLIP. J Cell Biol (2004) 166(3):369–80. doi: 10.1083/jcb.200401036 PMC2172264 15289496

[18] Chodorge M Züger S Stirnimann C Briand C Jermutus L Grütter MG. A Series of Fas Receptor Agonist Antibodies That Demonstrate an Inverse Correlation Between Affinity and Potency. Cell Death Differ (2012) 19(7):1187–95. doi: 10.1038/cdd.2011.208 PMC3374083 22261618

[19] Wang X Mathieu M Brezski RJ. IgG Fc Engineering to Modulate Antibody Effector Functions. Protein Cell (2018) 9(1):63–73. doi: 10.1007/s13238-017-0473-8 28986820 PMC5777978

[20] Kums J Nelke J Rüth B Schäfer V Siegmund D Wajant H. Quantitative Analysis of Cell Surface Antigen-Antibody Interaction Using Gaussia Princeps Luciferase Antibody Fusion Proteins. MAbs (2017) 9(3):506–20. doi: 10.1080/19420862.2016.1274844 PMC5384709 28095113

[21] Suffner J Hochweller K Kühnle M-C Li X Kroczek RA Garbi N. Dendritic Cells Support Homeostatic Expansion of Foxp3+ Regulatory T Cells in Foxp3.LuciDTR Mice. J Immunol (2010) 184(4):1810–20. doi: 10.4049/jimmunol.0902420 20083650

[22] Dahlhoff J Manz H Steinfatt T Delgado-Tascon J Seebacher E Schneider T. Transient Regulatory T-Cell Targeting Triggers Immune Control of Multiple Myeloma and Prevents Disease Progression. Leukemia (2021). 36(3):790–800 doi: 10.1038/s41375-021-01422-y 34584204 PMC8885410

[23] Beilhack A Schulz S Baker J Beilhack GF Wieland CB Herman EI. *In Vivo* Analyses of Early Events in Acute Graft-Versus-Host Disease Reveal Sequential Infiltration of T-Cell Subsets. Blood (2005) 106(3):1113–22. doi: 10.1182/blood-2005-02-0509 PMC1895168 15855275

[24] Bäuerlein CA Riedel SS Baker J Brede C Garrote A-LJ Chopra M. A Diagnostic Window for the Treatment of Acute Graft-Versus-Host Disease Prior to Visible Clinical Symptoms in a Murine Model. BMC Med (2013) 11:134. doi: 10.1186/1741-7015-11-134 23692886 PMC3665617

[25] Shaikh H Vargas JG Mokhtari Z Jarick KJ Ulbrich M Mosca JP. Mesenteric Lymph Node Transplantation in Mice to Study Immune Responses of the Gastrointestinal Tract. Front Immunol (2021) 12:689896. doi: 10.3389/fimmu.2021.689896 34381447 PMC8352558

[26] Yang Y Islam MS Hu Y Chen X. TNFR2: Role in Cancer Immunology and Immunotherapy. Immunotargets Ther (2021) 10:103–22. doi: 10.2147/ITT.S255224 PMC8071081 33907692

[27] Chopra M Riedel SS Biehl M Krieger S von Krosigk V Bäuerlein CA. Tumor Necrosis Factor Receptor 2-Dependent Homeostasis of Regulatory T Cells as a Player in TNF-Induced Experimental Metastasis. Carcinogenesis (2013) 34(6):1296–303. doi: 10.1093/carcin/bgt038 23385062

[28] Valencia X Stephens G Goldbach-Mansky R Wilson M Shevach EM Lipsky PE. TNF Downmodulates the Function of Human CD4+CD25hi T-Regulatory Cells. Blood (2006) 108(1):253–61. doi: 10.1182/blood-2005-11-4567 PMC1895836 16537805

[29] Chen X Subleski JJ Hamano R Howard OMZ Wiltrout RH Oppenheim JJ. Co-Expression of TNFR2 and CD25 Identifies More of the Functional CD4+FOXP3+ Regulatory T Cells in Human Peripheral Blood. Eur J Immunol (2010) 40(4):1099–106. doi: 10.1002/eji.200940022 PMC3096013 20127680

[30] Chen X Wu X Zhou Q Howard OMZ Netea MG Oppenheim JJ. TNFR2 Is Critical for the Stabilization of the CD4+Foxp3+ Regulatory T. Cell Phenotype in the Inflammatory Environment. J Immunol (2013) 190(3):1076–84. doi: 10.4049/jimmunol.1202659 PMC3552130 23277487

[31] Chen X Hamano R Subleski JJ Hurwitz AA Howard OMZ Oppenheim JJ. Expression of Costimulatory TNFR2 Induces Resistance of CD4+FoxP3- Conventional T Cells to Suppression by CD4+FoxP3+ Regulatory T Cells. J Immunol (2010) 185(1):174–82. doi: 10.4049/jimmunol.0903548 PMC6314668 20525892

[32] Nagar M Jacob-Hirsch J Vernitsky H Berkun Y Ben-Horin S Amariglio N. TNF Activates a NF-kappaB-Regulated Cellular Program in Human CD45RA- Regulatory T Cells That Modulates Their Suppressive Function. J Immunol (2010) 184(7):3570–81. doi: 10.4049/jimmunol.0902070 20181891

[33] Chen X Bäumel M Männel DN Howard OMZ Oppenheim JJ. Interaction of TNF With TNF Receptor Type 2 Promotes Expansion and Function of Mouse CD4+CD25+ T Regulatory Cells. J Immunol (2007) 179(1):154–61. doi: 10.4049/jimmunol.179.1.154 17579033

[34] Okubo Y Mera T Wang L Faustman DL. Homogeneous Expansion of Human T-Regulatory Cells *via* Tumor Necrosis Factor Receptor 2. Sci Rep (2013) 3:3153. doi: 10.1038/srep03153 24193319 PMC3818650

[35] Chen X Subleski JJ Kopf H Howard OMZ Männel DN Oppenheim JJ. Cutting Edge: Expression of TNFR2 Defines a Maximally Suppressive Subset of Mouse CD4+CD25+FoxP3+ T Regulatory Cells: Applicability to Tumor-Infiltrating T Regulatory Cells. J Immunol (2008) 180(10):6467–71. doi: 10.4049/jimmunol.180.10.6467 PMC2699949 18453563

[36] Punit S Dubé PE Liu CY Girish N Washington MK Polk DB. Tumor Necrosis Factor Receptor 2 Restricts the Pathogenicity of CD8(+) T Cells in Mice With Colitis. Gastroenterology (2015) 149(4):993–1005.e2. doi: 10.1053/j.gastro.2015.06.004 26072395 PMC4841683

[37] Blüml S Binder NB Niederreiter B Polzer K Hayer S Tauber S. Antiinflammatory Effects of Tumor Necrosis Factor on Hematopoietic Cells in a Murine Model of Erosive Arthritis. Arthritis Rheumatol (2010) 62(6):1608–19. doi: 10.1002/art.27399 20155834

[38] Madsen PM Motti D Karmally S Szymkowski DE Lambertsen KL Bethea JR. Oligodendroglial TNFR2 Mediates Membrane TNF-Dependent Repair in Experimental Autoimmune Encephalomyelitis by Promoting Oligodendrocyte Differentiation and Remyelination. J Neurosci (2016) 36(18):5128–43. doi: 10.1523/JNEUROSCI.0211-16.2016 PMC4854972 27147664

[39] Fischer R Maier O Siegemund M Wajant H Scheurich P Pfizenmaier K. A TNF Receptor 2 Selective Agonist Rescues Human Neurons From Oxidative Stress-Induced Cell Death. PLoS One (2011) 6(11):e27621. doi: 10.1371/journal.pone.0027621 22110694 PMC3215731

[40] Ortí-Casañ N Wu Y Naudé PJW De Deyn PP Zuhorn IS Eisel ULM. Targeting TNFR2 as a Novel Therapeutic Strategy for Alzheimer’s Disease. Front Neurosci (2019) 13:49. doi: 10.3389/fnins.2019.00049 30778285 PMC6369349

[41] Pierini A Strober W Moffett C Baker J Nishikii H Alvarez M. TNF-α Priming Enhances CD4+FoxP3+ Regulatory T-Cell Suppressive Function in Murine GVHD Prevention and Treatment. Blood (2016) 128(6):866–71. doi: 10.1182/blood-2016-04-711275 PMC4982455 27365424

[42] Leclerc M Naserian S Pilon C Thiolat A Martin GH Pouchy C. Control of GVHD by Regulatory T Cells Depends on TNF Produced by T Cells and TNFR2 Expressed by Regulatory T Cells. Blood (2016) 128(12):1651–9. doi: 10.1182/blood-2016-02-700849 27506541

[43] Fontaine V Mohand-Said S Hanoteau N Fuchs C Pfizenmaier K Eisel U. Neurodegenerative and Neuroprotective Effects of Tumor Necrosis Factor (TNF) in Retinal Ischemia: Opposite Roles of TNF Receptor 1 and TNF Receptor 2. J Neurosci (2002) 22(7):RC216. doi: 10.1523/JNEUROSCI.22-07-j0001.2002 11917000 PMC6758303

[44] Tam EM Fulton RB Sampson JF Muda M Camblin A Richards J. Antibody-Mediated Targeting of TNFR2 Activates CD8+ T Cells in Mice and Promotes Antitumor Immunity. Sci Transl Med (2019) 11(512):eaax0720. doi: 10.1126/scitranslmed.aax0720 31578241

[45] de Kivit S Mensink M Hoekstra AT Berlin I Derks RJE Both D. Stable Human Regulatory T Cells Switch to Glycolysis Following TNF Receptor 2 Costimulation. Nat Metab (2020) 2(10):1046–61. doi: 10.1038/s42255-020-00271-w 32958937

[46] He X Landman S Bauland SCG van den Dolder J Koenen HJPM Joosten I. A TNFR2-Agonist Facilitates High Purity Expansion of Human Low Purity Treg Cells. PLoS One (2016) 11(5):e0156311. doi: 10.1371/journal.pone.0156311 27224512 PMC4880213

[47] Ban L Kuhtreiber W Butterworth J Okubo Y Vanamee ÉS Faustman DL. Strategic Internal Covalent Cross-Linking of TNF Produces a Stable TNF Trimer With Improved TNFR2 Signaling. Mol Cell Ther (2015) 3:7. doi: 10.1186/s40591-015-0044-4 26266038 PMC4531505

[48] Maier O Fischer R Agresti C Pfizenmaier K. TNF Receptor 2 Protects Oligodendrocyte Progenitor Cells Against Oxidative Stress. Biochem Biophys Res Commun (2013) 440(2):336–41. doi: 10.1016/j.bbrc.2013.09.083 24076392

[49] Joedicke JJ Myers L Carmody AB Messer RJ Wajant H Lang KS. Activated CD8+ T Cells Induce Expansion of Vβ5+ Regulatory T Cells *via* TNFR2 Signaling. J Immunol (2014) 193(6):2952–60. doi: 10.4049/jimmunol.1400649 PMC4157120 25098294

[50] Gerald MJ Bracchi-Ricard V Ricard J Fischer R Nandakumar B Blumenthal GH. Continuous Infusion of an Agonist of the Tumor Necrosis Factor Receptor 2 in the Spinal Cord Improves Recovery After Traumatic Contusive Injury. CNS Neurosci Ther (2019) 25(8):884–89354. doi: 10.1111/cns.13125 30941924 PMC6630008

[51] Fischer R Sendetski M Del Rivero T Martinez GF Bracchi-Ricard V Swanson KA. TNFR2 Promotes Treg-Mediated Recovery From Neuropathic Pain Across Sexes. Proc Natl Acad Sci USA (2019) 116(34):17045–50. doi: 10.1073/pnas.1902091116 PMC6708347 31391309

[52] Pardridge WM. Kinetics of Blood-Brain Barrier Transport of Monoclonal Antibodies Targeting the Insulin Receptor and the Transferrin Receptor. Pharmaceut (Basel) (2021) 15(1):3. doi: 10.3390/ph15010003 PMC8778919 35056060

[53] Bajracharya R Caruso AC Vella LJ Nisbet RM. Current and Emerging Strategies for Enhancing Antibody Delivery to the Brain. Pharmaceutics (2021) 13(12):2014. doi: 10.3390/pharmaceutics13122014 34959296 PMC8709416

