# Corrigendum: A TNFR2-specific TNF fusion protein with improved *in vivo* activity

**DOI:** 10.3389/fimmu.2023.1352525

**Published:** 2023-12-18

**Authors:** Juan Gamboa Vargas, Jennifer Wagner, Haroon Shaikh, Isabell Lang, Juliane Medler, Mohamed Anany, Tim Steinfatt, Josefina Peña Mosca, Stephanie Haack, Julia Dahlhoff, Maike Büttner-Herold, Carolin Graf, Estibaliz Arellano Viera, Hermann Einsele, Harald Wajant, Andreas Beilhack

**Affiliations:** ^1^Interdisciplinary Center for Clinical Research Laboratory, Department of Internal Medicine II, University Hospital Würzburg, Würzburg, Germany; ^2^Graduate School of Life Sciences, Würzburg University, Würzburg, Germany; ^3^Division of Molecular Internal Medicine, Department of Internal Medicine II, University Hospital Würzburg, Würzburg, Germany; ^4^Department of Microbial Biotechnology, Institute of Biotechnology, National Research Center, Giza, Egypt; ^5^Department of Nephropathology, Friedrich-Alexander-Universität Erlangen-Nürnberg, Erlangen, Germany

**Keywords:** agonist, GvHD, regulatory T cells, serum retention, TNF, TNFR2

**Incorrect Affiliation**

In the published article, there was an error in affiliation “4”. Instead of “Department of Microbial Biotechnology, Institute of Biotechnology, Giza, Egypt”, it should be “Department of Microbial Biotechnology, Institute of Biotechnology, National Research Center, Giza, Egypt”.

The authors apologize for this error and state that this does not change the scientific conclusions of the article in any way. The original article has been updated.

