# Supplementary material for: A TNFR2-Specific TNF Fusion Protein With Improved In Vivo Activity
Source: Front Immunol. 2022 Jun 13;13:888274. doi: 10.3389/fimmu.2022.888274 (PMC9234581; doi:10.3389/fimmu.2022.888274)
Supplement: Supplementary file 1 [file DataSheet_1.pdf]

Supplementary Material

A TNFR2-specific TNF fusion protein with improved in vivo activity

Juan Gamboa Vargas, Jennifer Wagner, Haroon Shaikh, Isabell Lang, Juliane Medler, Mohamed Anany, Tim Steinfatt, Josefina Peña Mosca, Stephanie Haack, Julia Dahlhoff, Maike Büttner-Herold, Carolin Graf, Estibaliz Arellano Viera, Hermann Einsele, Harald Wajant, Andreas Beilhack

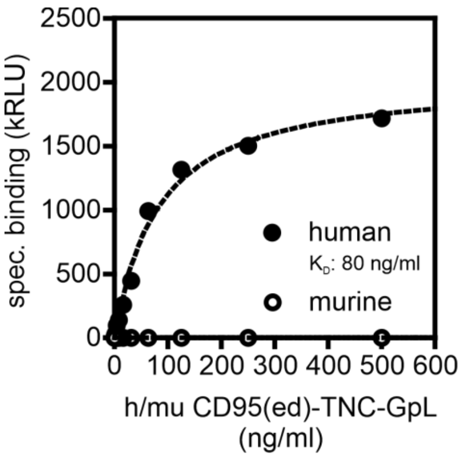

Supplementary Figure S1. NewSTAR2 and as control an irrelevant antibody were immobilized on 96-well plates. Trimeric GpL fusion proteins of the extracellular domain of CD95 (hCD95(ed)-TNC-GpL and muCD95(ed)-TNC-GpL) were added pairwise with the indicated concentrations. After removal of unbound proteins, GpL activities were measured. Specific binding values were obtained by subtraction of the unspecific binding values (irrelevant antibody) from the total binding values (NewSTAR2). Data were analyzed with the “nonlinear regression to a one-site specific binding curve” function of the GraphPad Prism5 software.

AA sequence of the heavy chain of irrIgG1(N297A)-HC:sc(mu)TNF80 / NewSTAR2

*MNFGFSLIFLVLVLKGVQCEVKLVPRQL***DYKDDDDK**ELQLQLQESGPGLVKPSETLSLTCTVSGASISANSYYGVWVRQSP GKGLEWVGSIAYRGNSNSGSTYYNPSLKSRATVSVDTSKNQVSLRLTSVTAADTALYYCARRQLLDDGTGYQWAAFDVWGQ GTMVTVSSGSSSASTKGPSVFPLAPSSKSTSGGTAALGCLVKDYFPEPVTVSWNSGALTSGVHTFPAVLQSSGLYSLSSVV TVPSSSLGTQTYICNVNHKPSNTKVDKKVEPKSCDKTHTCPPCPAPELLGGPSVFLFPPKPKDTLMISRTPEVTCVVVDVS HEDPEVKFNWYVDGVEVHNAKTKPREEQYASTYRVVSVLTVLHQDWLNGKEYKCKVSNKALPAPIEKTISKAKGQPREPQV YTLPPSRDELTKNQVSLTCLVKGFYPSDIAVEWESNGQPENNYKTTPPVLDSDGSFFLYSKLTVDKSRWQQGNVFSCSVMH EALHNHYTQKSLSLSPGKEFEFTRDKPVAHVVANHQVEEQLEWLSQRANALLANGMDLKDNQLVVPADGLYLVYSQVLFKG QGCPDYVLLTHTVSRFAISYQEKVNLLSAVKSPCPKDTPEGAELKPWYEPIYLGGVFQLEKGDQLSAEVNLPKYL**N**F**R**ESG QVYFGVIALGGGSGGGSGGGSGGGSDKPVAHVVANHQVEEQLEWLSQRANALLANGMDLKDNQLVVPADGLYLVYSQVLFK GQGCPDYVLLTHTVSRFAISYQEKVNLLSAVKSPCPKDTPEGAELKPWYEPIYLGGVFQLEKGDQLSAEVNLPKYL**N**F**R**ES GQVYFGVIALGGGSGGGSGGGSGGGSDKPVAHVVANHQVEEQLEWLSQRANALLANGMDLKDNQLVVPADGLYLVYSQVLF KGQGCPDYVLLTHTVSRFAISYQEKVNLLSAVKSPCPKDTPEGAELKPWYEPIYLGGVFQLEKGDQLSAEVNLPKYL**N**F**R**E SGQVYFGVIAL

Leader, Flag-tag, antiCD95(E09)-VH, heavy constant (N297A), sc(mu)TNF**80**, linker, cloning-related insertions

AA sequence of the light chain of irrIgG1(N297A)-HC:sc(mu)TNF80 / NewSTAR2

*MNFGFSLIFLVLVLKGVQCEVKLVPRQL***DYKDDDDK**ELQSVLTQPPSVSEAPRQTVTISCSGNSFNIGRYPVNWYQQLPGK APKLLIYYNNLRFSGVSDRFSGSKSGTSASLAIRDLLSEDEADYYCSTWDDTLKGWVFGGGTKVTVLGGSEIKRTVAAPSV FIFPPSDEQLKSGTASVVCLLNNFYPREAKVQWKVDNALQSGNSQESVTEQDSKDSTYSLSSTLTLSKADYEKHKVYACEV THQGLSSPVTKSFNRGEC

Leader, Flag-tag, antiCD95(E09)-VL, light constant, cloning-related insertions

Supplementary Figure S2. Amino acid sequences of the heavy and light chains of NewSTAR2. The two point mutations in the muTNF protomers conferring TNFR2 specificity are shown in red, the mutation in the heavy chain interfering with FcγR binding is shown in grey.

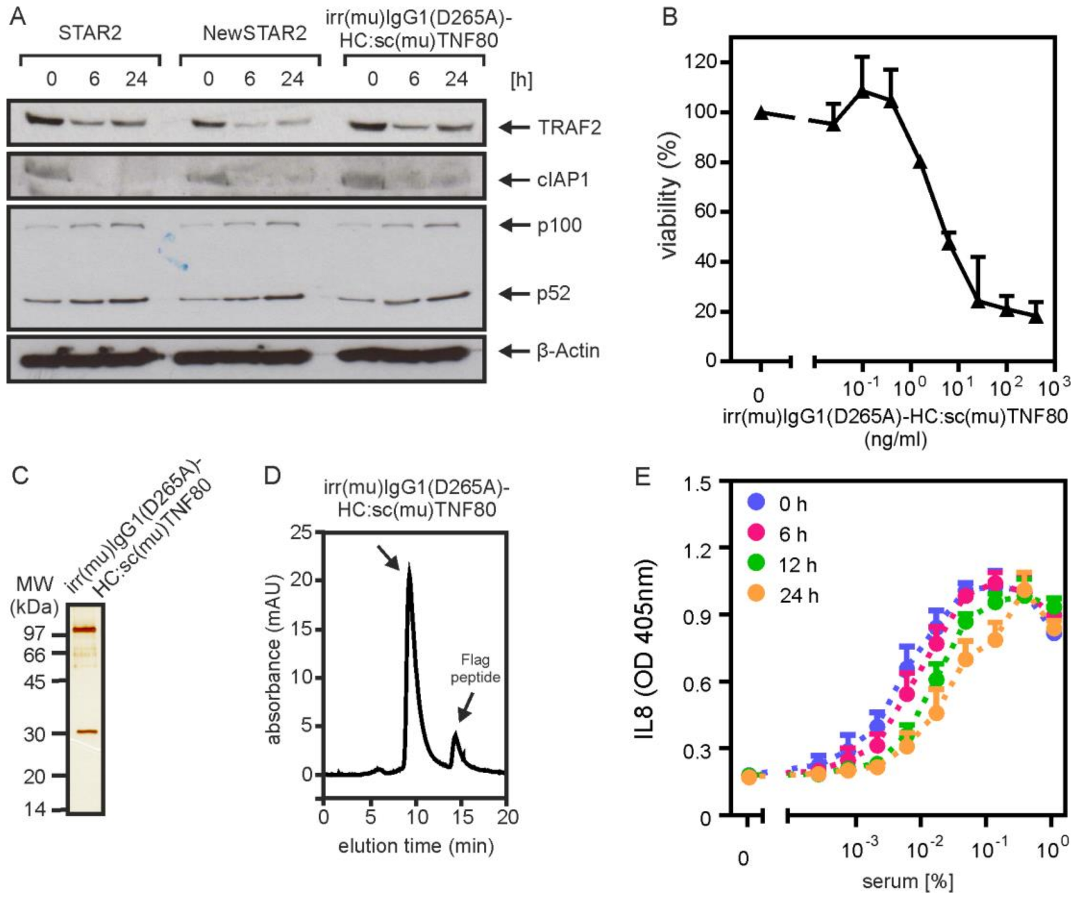

Supplementary Figure S3. Characterization and purification of a NewSTAR2 variant with murine IgG1 scaffold. (**A**) Macrophages differentiated from Hoxb8 immortalized murine MPCs were challenged overnight with 200 ng/ml of STAR2, NewSTAR2 and the murine IgG1 variant of NewSTAR2 (irr(mu)IgG1(D265A)-HC:sc(mu)TNF80) and total cell lysates were analyzed by western blotting. (**B**) Macrophages were stimulated for 36 h with the indicated concentrations of irr(mu)IgG1(D256A)-HC:sc(mu)TNF80) in the presence of 20 µM zVAD-fmk and finally viability was evaluated. (**C,D**) irr(mu)IgG1(D265A)-HC:sc(mu)TNF80) was purified by anti-Flag affinity chromatography and analyzed by SDS-PAGE (C) and gel filtration (D). (**E**) Three mice were i.v. injected with irr(mu)IgG1(D265A)-HC:sc(mu)TNF80) (100 µg). Blood was taken after the indicated imes and used to stimulate HT1080-Bcl2-TNFR2. IL-8 production was evaluated next day to quantify TNFR2 engagement.

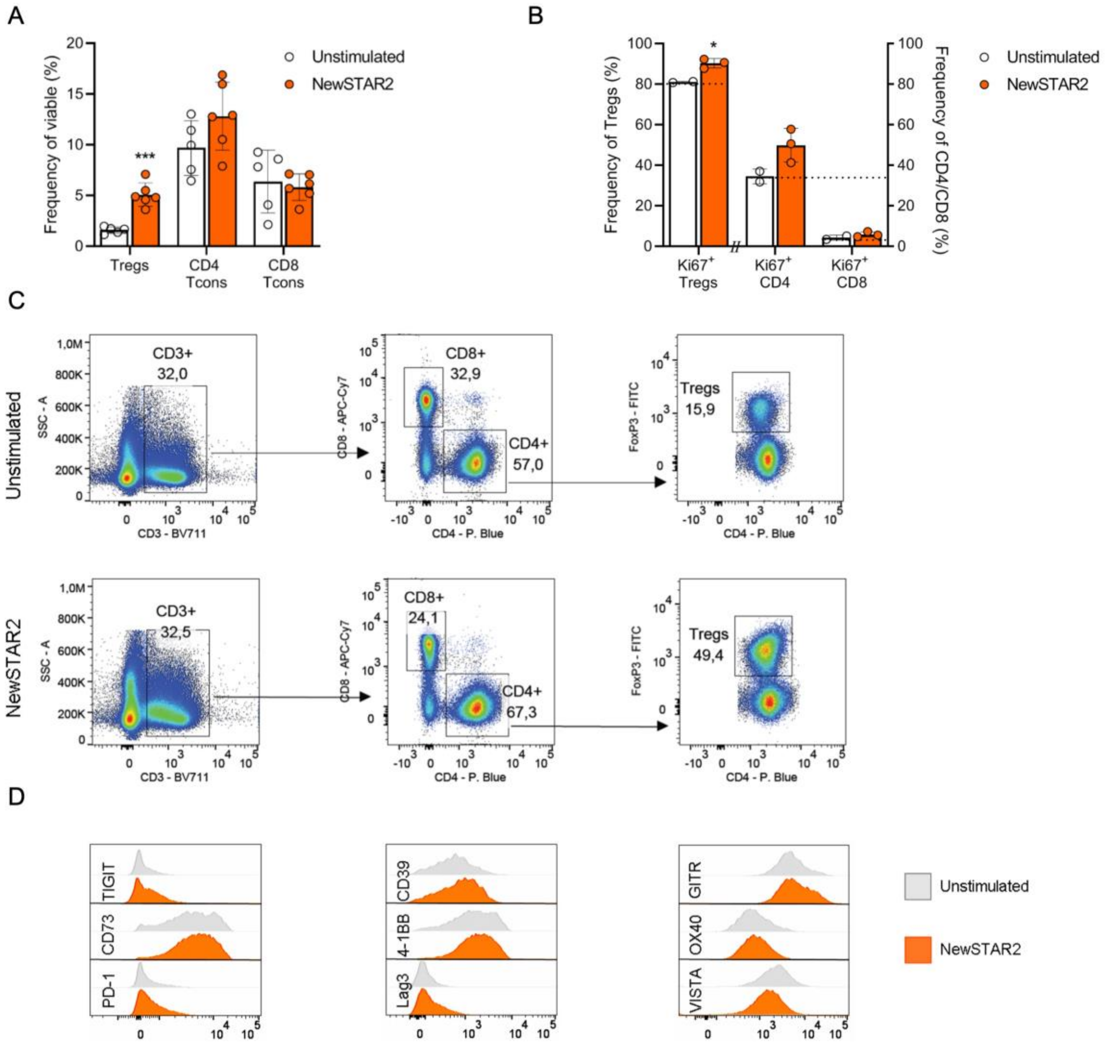

Supplemental Figure S4. NewSTAR2 specifically expands Tregs but not conventional T cell (Tcon) populations. **(A)** Frequencies of viable Treg and Tcon populations from splenocytes isolated 4 days after in vivo stimulation with NewSTAR2. Splenic cells from untreated animals served as control (unsitmulated). **(B)** Frequencies of proliferating Tregs and Tcons described by their intracellular Ki67 expression. Proliferating Tregs ($CD4^+FoxP3^+Ki67^+$) shown as frequencies from the total Treg population, while Tcons values are shown as frequencies of viable cells. Each data point represents averaged values of triplicate measurements from one whole spleen +/- S.D. *$p \leq 0.05$, ** $p \leq 0.01$, ***$p \leq 0.001$, two-tailed unpaired Student's *t*-test. (**C**) Representative dot plots of splenocytes samples 4 days after in vivo stimulation with 140 µg of NewSTAR2 or control antibody (= unstimulated). Populations were obtained after selecting lymphocytes by size exclusion, singlets and viable cells. (**D**) Representative histograms of the normalized mean fluorescence intensity of the shown markers expressed by the Treg population as defined in (C). Histograms of splenic Tregs after NewSTAR2 treatment in orange; splenic Tregs after control antibody treatment in light grey.

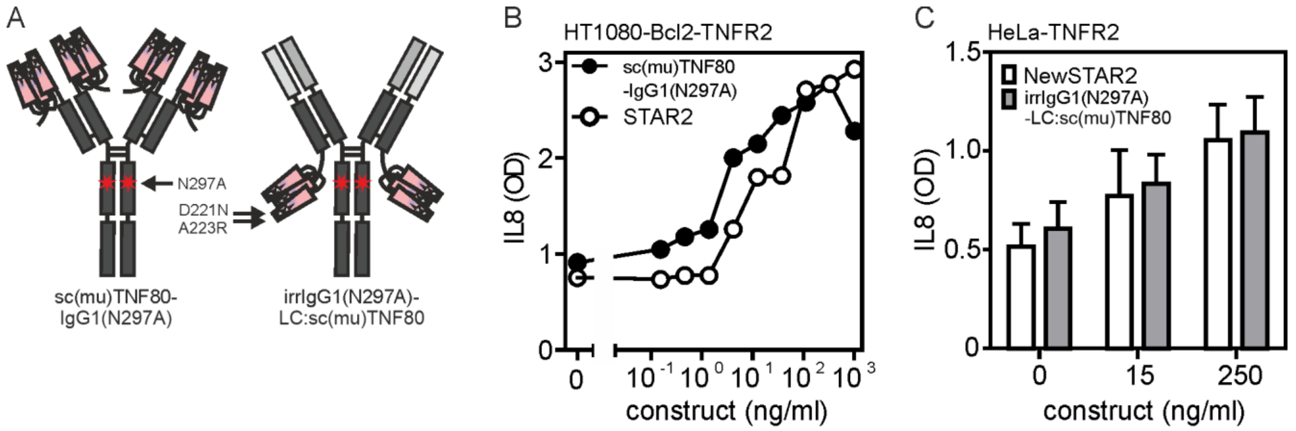

Supplemental Figure S5. sc(mu)TNF80-IgG1(N297A) and STAR2 and irrIgG1(N297A)-LC:sc(mu)TNF80 and NewSTAR2 are comparably active. (**A**) Domain architecture of sc(mu)TNF80-IgG1(N297A) and irrIgG1(N297A)-LC:sc(mu)TNF80. **(B)** HT1080-Bcl2-TNFR2 cells were stimulated overnight as indicated with STAR2 and sc(mu)TNF80-IgG1(N297A). Next day, cell culture supernatants were analyzed for IL8 production by ELISA. Shown is one of three representative experiments. (**C**) HeLa-TNFR2 cells were stimulated overnight with 15 and 250 ng/ml of NewSTAR2 and irrIgG1(N297A)-LC:sc(mu)TNF80. Next day, cell culture supernatants were again analyzed for IL8 production by ELISA. Shown are the pooled results from three independent experiments +/- SD.
